# Tumor immunology meets oncology (TIMO) XVII, April 20–22 2023 in Halle/Saale, Germany

**DOI:** 10.1007/s00262-023-03553-w

**Published:** 2023-10-23

**Authors:** Barbara Seliger

**Affiliations:** 1 Institute for Translational Immunology, Brandenburg Medical School, Theodor Fontane”, Hochstraße 29, 14770 Brandenburg, Germany; 2 Medical Faculty, Martin-Luther-University Halle-Wittenberg, Magdeburger Straße 02, 06112 Halle (Saale), Germany; 3 Fraunhofer Institute for Cell Therapy and Immunology, 04103 Leipzig, Germany

**Keywords:** Immune escape, Immunotherapy, Combination therapies, Immune resistance, Tumor microenvironment, TIMO 2023

## Abstract

During the TIMO meeting 2023, national and international scientists as well as clinicians gave novel insights as well as perspectives into basic and translational tumor immunology. https://dgfi.org/arbeitskreise/ak-tumorimmunologie/meeting/

## Introduction

The yearly “Tumor immunology meets oncology (TIMO)” meeting was held from the 20th to the 22nd of April 2023 at the Steintor Variety in Halle, Germany and consists of a workshop at the first day followed by the main symposium for two days.

The annual TIMO meeting comprised national and international experts from basic and translational science as well as clinicians, which allowed discussing the current and future developments in tumor immunology, immunotherapy and immune oncology. During the last years, an increased understanding why patients with certain tumor types did respond to immunotherapy or were non-responders has been obtained. Based on the increased knowledge of the tumor immune surveillance and escape, novel treatment concepts with new agents and/or combinations were developed to prevent or overcome immune resistances, which gave the participants the opportunity to discuss their own research, but also to interact and network with scientists all over the world. The goal of this years’ symposium was to present the latest research findings in basic immunology as well as in the translation of this knowledge into clinical application, such as the design and implementation of novel innovative immunotherapies alone or in combinations with other therapeutic options in order to increase treatment efficacy and to reduce side effects. Thus, the symposium covered a number of preclinical, translational and clinical topics encompassing both solid as well as hematologic malignancies, which were divided into 5 different sessions building on each other. The first session addressed basic immunologic questions, such as tumor immune evasion including the role of microRNAs (miRNAs) and metabolites and the cellular composition of the tumor microenvironment (TME) in this process, their clinical relevance as well as the implementation of different nanoparticles for efficient transduction of immune-relevant molecules into tumor cells and/or different immune cell subpopulations. The second topic covered the identification of biomarkers used for prognosis, prediction of therapy responses and resistances using distinct approaches. This included next generation sequencing (NGS) to identify neoantigens, monitoring of tissues and/or blood with various high-end technologies, such as multiplex immunohistochemistry (IHC), imaging mass cytometry (IMC), multicolour flow cytometry, mass cytometry (CyToF), RNA sequencing (RNAseq), single cell RNA sequencing (scRNAseq) and application of artificial intelligence (AI) approaches. The third and fourth session focussed on the implementation of different immunotherapies, combination of immunotherapies with other treatment options as well as the development of novel approaches or the improvement of protocols based on the molecular or immunologic characterization of the tumors. As established over the years, TIMO XVI started with a workshop to promote young investigators followed by a symposium program on the topics described above including novel application strategies and innovative therapeutic strategies, which demonstrated the power of the new technologies and the use of bioinformatics tools.

The first session of the symposium on basic tumor immunological questions started with a talk of the organiser Barbara Seliger (Medical School “Theodor Fontane”, Brandenburg and Medical Faculty, Martin Luther University Halle-Wittenberg, Germany). She summarized novel insights into different immune evasion strategies of cancer cells with a major focus on the downregulation or loss of the HLA class I antigen processing machinery (APM) components. These were frequently found in different tumor types and were associated with a reduced survival and resistance of cancer patients to different immunotherapeutic approaches. The underlying mechanisms of such HLA class I alterations could occur at distinct levels including posttranscriptional regulation, which were often mediated by a deregulation of immune modulatory miRNAs (miRNAs) targeting immune-relevant molecules. These miRNAs identified by the Seliger team using a miRNA enrichment technology in combination with RNAseq were shown to control different HLA class I APM components, as well as the immune checkpoints (ICPs) HLA-G and programmed death ligand 1 (PD-L1) and their expression, was associated with the disease outcome and the architecture of the TME. Furthermore, other factors, like e.g. members of the small leucine-rich proteoglycan family, like biglycan (BGN), were downregulated in tumors and oncogene-transformed cells with low HLA class I surface antigen expression when compared to corresponding non-neoplastic controls. Restoration of BGN expression not only reduced migration and proliferation of tumor cells, but also directly increased HLA class I APM component expression. This was partially due to a downregulation of the oncogenic miR-21, which correlated with an increased overall survival (OS) of patients. Thus, novel approaches for treatment of tumor patients were suggested by changing the extracellular matrix (ECM) composition and modulating miRNAs, but the strategy how to implement both strategies, e.g. the optimal delivery system, is still under debate. In sum, the immune escape mechanisms of tumors were diverse and ranged from classical and non-classical HLA class I antigens, ICPs and ECM components and were based on altered expression patterns of structural abnormalities and distinct regulatory processes of the molecules.

Achim Aigner from the Clinical Pharmacology of Leipzig University, Leipzig, Germany talked about nanoparticle-based delivery of nucleic acids into tumor and immune cells to enhance/ deliver anti-tumor responses. These drug candidates included siRNA, mRNA, but also miRNA mimics, anti-miRNAs as well as circular miRNAs with the advantage of their direct action on target (m)RNAs, specificity and efficacy, as well as the possibility to target otherwise, undruggable genes. However, disadvantages were their instability mediated by degradation, rapid excretion, poor tissue penetration and in vivo delivery as well as the lack of approaches for targeted delivery of RNA molecules into diseased tissues. So far, a number of delivery strategies for RNAs in vivo were described, such as different nanocarriers, like polymer-based nanocarriers, lipid/liposomal systems, different drug conjugates and modified nanoparticles, which reduced their non-specific interaction, but increased their efficacy. Polymeric nanocarriers based on polyethylenimines (PEIs) could be efficiently applied for small interfering RNA (siRNA) delivery into different tumor models including patient-derived tumor xenografts (PDXs) by systemic injection, thereby reducing tumor size and mediating gene knockdown. Next to siRNAs, miRNAs and anti-miRNAs were delivered via tyrosine-modified nanoparticles, which also increased the transfection efficacy in 3D tissue models, primary mesenchymal stromal cells and different immune cells, representing e.g. macrophages or pro-monocytes. This talk gave insights into the efficacy and specificity of nucleic acid delivery with low side effects.

Vincenzo Bronte (The Veneto Institute of Oncology, Padova, Italy) reported about neutrophil extracellular traps (NETs), which are networks of extracellular strings primarily composed of DNA from neutrophils and are able to modulate the immune system. He addressed the role of NETs in pancreatic ductal adenocarcinoma (PDAC), where these structures restrained anti-tumor T cell activity. Analyzing myeloid pro-tumor functions, a different gene profile characterized by distinct molecular features, such as pSTAT3 and arginine-1 (ARG1), was found in the immune suppressive monocytes of PDAC. In addition, the ARG1 blockade in tumor mouse models favoured tumor rejection following adoptive cell therapy (ACT). However, it is noteworthy that the ARG1 biology is distinct in mice and humans thereby restricting the clinical translation of experimental results. In humans, ARG1 is stored in tertiary granules as an inactive protein and L-arginine hydrolysis takes place extracellularly. NETs create a microdomain, where cathepsin S cleaves human ARG1 into different isoforms with increased enzymatic activity at physiological pH, but its role in immune suppression and mechanisms of action were not clearly defined. So far, V. Bronte demonstrated that NETs suppressed T cell proliferation by an ARG1-dependent mechanisms, while neutralizing antibodies (Abs) targeting ARG1 or preventing cathepsin S cleavage restored T cell activity. The TME of PDAC biopsies was enriched in NETs and ARG1 and their neutralization increased the functional status of the infiltrating lymphocytes in PDAC, which was enhanced in the presence of ICPi and ACT.

Qing Yang (School of Life Sciences, Fudan, China) delivered a talk on the relevance of indolamine 2,3-deoxygenase (IDO), which is a major player of the tryptophan metabolism and exerts immune suppressive properties by activation of the aryl hydrocarbon receptor (AhR) pathway, in the immune escape of tumors and regarding its clinical relevance. IDO1 impaired NK cell function by a downregulation of the activating receptor natural killer group 2D (NKG2D) on NK cells as well as the expression of the ligand of NKG2D (NKG2D-L) in non-small lung carcinoma (NSCLC) cells. The implementation of an IDO inhibitor reverted the tryptophan depletion, promoted T cell effector activation, inhibited Treg proliferation and improved NK cell function. However, the effect of IDO inhibitors is currently controversially discussed in cancer immunotherapy and so far, no IDO inhibitor got clinical approval. Thus, more specific IDO1 inhibitors or therapy combinations, a better clinical trial design and biomarkers for monitoring therapy response were urgently needed. In glioblastoma, IDO1 expression was increased and negatively correlated with prognosis and patients’ survival. The combination of IDO1/TDO inhibitor blocked angiogenesis and tube formation in glioma and also in PDAC. In sum, Q. Yang reported about improved IDO1 inhibitors/ combinations with in vivo activity, which are currently developed for their clinical application.

Noel de Miranda from the Leiden University Medical Center, Leiden, The Netherlands, presented interesting data about new players for immunotherapeutic approaches in colorectal cancer (CRC) with focus on *γδ* T cells. This was based on the fact that mismatch repair (MMR)-deficient (MMR-d) CRC had often lost HLA class I expression when compared to MMR-proficient (MMR-p) CRCs. Different *γδ* T cell subpopulations identified by single cell RNA-sequencing (scRNAseq) were associated with their functional status regarding activation, cytotoxicity, proliferative capacity as well as the expression of different activating and inactivating ICP receptors. These *γδ* T cells were cytotoxic against HLA class I-negative CRCs and in particular enriched in HLA class I-negative MMR-d cancers. In order to determine their relevance in immunotherapy, their frequency in tissues was analysed demonstrating an enrichment of *γδ* T cells in *β*_2_-microglobulin (*β*_2_-*m*)-negative MMR-d CRC treated with ICPi, mainly of the *γδ*1 phenotype. Interestingly, anti-PD-1 treatment increased the cytotoxic activity of *γδ* T cells against HLA class I-negative cancer cells. Next to *γδ* T cells, the functional role of innate lymphocyte cells (ILCs) in response to ICPi was investigated identifying an ILC-1-like cellular subset, which had a distinct transcriptional activity when compared to NK cells.

Using a DMBA-induced MPA-accelerated (M/D) breast cancer (BC) preclinical model, Aitziber Buqué Martinez (Weill Cornell Medical Center, New York, USA) gave insights how to modulate the TME to improve radiation therapy (RT) and endocrine-based therapies. This is an important topic, since resistance to immune checkpoint blockade (ICB) is still a major obstacle for hormone receptor (HR)-positive BC and novel strategies are urgently needed to improve anti-tumor responses. A. Buqué Martinez presented data using female mice bearing M/D-driven BC treated with either anti-PD1 or CDK4/6 inhibitors, which lead to an increased survival, while a combination with RT enhanced this effect. The transcriptional profile analysis of M/D-driven tumors compared with non-tumor mammary gland tissue resulted in a reduced presentation of pathways involved in the immune system activity, but an overrepresentation of pathways associated with proliferation, pointing to a strong immune surveillance activity during the oncogenic process. These findings were validated by depleting specific immune cell populations with NK cells as the major players of early immune surveillance during M/D-driven HR-positive oncogenic process. This immune surveillance was influenced by nutritional interventions, such high fat diet, which accelerated the oncogenic process, or supplements targeting the nicotinamide adenine dinucleotide (NAD)^+^, which decreased the onset of the disease by boosting the immune system. Targeting the tryptophan catabolism pathway in M/D-driven tumors by 3-hydroxy-L-kynurenamine (3-HKA) delayed oncogenesis and extended the overall survival (OS) of mice. Thus, targeting of the NAD^+^ levels affected both tumor growth and the activity of immune cells. A NAM-enriched diet directly synergized with the ICB against HR-positive BC, while 3-HKA had a significant anti-tumor activity by directly affecting the tumor cell proliferation independent of the immune system. Therefore, 3-HKA treatment in combination with immune stimulatory therapies, such as RT or CDK4/6 inhibitors, may result in a superior tumor control at least in mice.

In this context, Lorenzo Galluzzi from the Weill Cornell Medical College in New York, USA, talked about the use of RT to improve the efficacy of immunotherapy with specific respect to response, duration and rate. On the one hand, Galluzzi provided preclinical evidence supporting the ability of low-dose whole-body RT delivered shortly before infusion of CD19-targeting chimeric antigen receptor (CAR) T cells to extent their efficacy against human CD19^+^ acute lymphoblastic leukemia cells growing in immunodeficient mice. On the other hand, Galluzzi discussed the impact of RT delivered focally according to different doses and fractionation schedules on tumor control in an immunocompentent model of HR-positive BC with unique translational features. In this setting, RT was very efficient at controlling tumor growth and extending the overall survival of BC-bearing mice, but did not synergize with an ICPi targeting PD-1, with a potential impact of stromal regulatory T cells (Treg). Moreover, RT fractionation schedules appeared to dictate local vs systemic disease control by RT in this model. Thus, conventional RT schedule may need to be adapted to develop efficient combinations with immunotherapy, in particular regarding fractionation schedules, target volumes and timing of administration.

In addition, Sandra Demaria from the Weill Cornell Medical College in New York, USA, addressed the enhancement of response to immunotherapy by RT based on combining RT with cytotoxic T lymphocyte-associated protein 4 (CTLA-4) blockade in lung cancer. Employing in vivo murine models, resistance mechanisms and combination treatments were studied. The combination of RT with anti-CTLA-4 did lead to an increase in the clonality of intra tumoral T cells. In addition, single cell analysis revealed that this combinatorial treatment resulted in a reduced frequency of Treg, but an increased frequency of Th1-like CD4^+^ T cells and effector memory and an early activation of CD8^+^ T cells. Furthermore, neither additional targeting of PD-1 and LAG3 expressed by exhausted T cells nor of GITR and OX40 highly expressed by Treg did improve responses. In contrast, treatment with an agonistic CD40 Ab resulted in tumor responses in the majority of tumor-bearing mice, an effect that was dependent on CD8^+^ T cells. Additional analyses showed an increased activation of dendritic cells (DC) associated with an increased tumor-specific T cell priming. However, combinatorial approaches of RT and ICP have to be further investigated to improve patients’ outcome.

Ofer Mandelboim (The Hebrew University of Jerusalem, Jerusalem, Israel) linked the presence of *Fusobacterium (F.) nucleatum* to the progression of different tumors. He demonstrated that *F. nucleatum* enhanced the proliferation of tumor cells, established a tumor-promoting environment by suppressing the accumulation of TIL, induced chemotherapy resistance, disease recurrence and poor patients’ prognosis. In addition, the anti-tumoral activity of TIL and NK cells was inhibited by ICP, like TIGIT and CEACAM1. Furthermore, due to the overabundance of *F. nucleatum* in tumors, it could be employed for the detection of tumors, despite screening capabilities on large cohorts of tumor samples have not yet been established. Based on the data, *F. nucleatum* could be used as a platform for targeting primary tumors and metastasis, such as targeting tumor colonized fusobacteria with bacteriophages engineered to express anti-cancer payloads.

Mascha Binder, a clinician scientist and head of Medical Oncology at the University Hospital in Basel, Switzerland, provided a comprehensive overview of the combination of immune checkpoint inhibitors (iCPi) with chemotherapy and targeted therapy for the treatment of gastrointestinal (GI) tumors. Currently, GI cancers are managed with different ICPi agents that target the PD-1/ PD-L1 axis and CTLA-4. Despite the progress, determining the most effective drug combinations for first-line therapy and adopting a multi-targeting approach remain unresolved challenges and the identification of biomarkers for monitoring treatment responses is critical to screen and track disease progression and therapy outcomes. In addition to biomarkers, the optimization of combinatorial approach requires careful consideration of the drugs used and their mode of administration, including determining the optimal dosage that may be different from previous chemotherapy-only protocols that relied on maximum tolerated doses. In conclusion, Mascha Binder's presentation shed light on the potential of combining iCPi with chemotherapy and targeted therapy for GI tumor treatment. Nevertheless, addressing the challenges of selecting optimal drug combinations, identifying relevant biomarkers and refining treatment administration is of paramount importance in advancing this field and improving patients’ outcomes.

Sergio Rutella (John van Geest Cancer Research Centre, Nottingham, Great Britain) gave a talk on the clinical implications of T cell dysfunction in acute myeloid leukaemia (AML). He specifically focussed on gene signatures of immune effector dysfunction (IED) and their correlation with established AML prognosticators, including mutations of *RUNX1* and *TP53*, and with lack of response to both standard-of-care chemotherapy and pembrolizumab immunotherapy in patients with chemotherapy-refractory disease. He also showed that IED gene sets predicted a poor response to ICPi in renal cell carcinoma, pointing to a broader role of T cell dysfunction in mediating immunotherapy resistance beyond AML. He leveraged computational biology approaches to unveil compositional differences in the AML TME of patients who did not respond to ipilimumab immunotherapy, such as a higher frequency of monocytic cell types, type 2 DC and cellular neighbourhoods enriched with leukaemia stemness gene programs. The IED signature identified in this work could therefore, predict and define the outcome of ICPi treatment in AML patients and thus could be used for stratification and selection of AML patients for personalized immunotherapies.

Hubert Hackl (Medical University Innsbruck, Innsbruck, Austria) presented a bioinformatics analysis demonstrating the vulnerability of combinations of immunotherapy in high-grade serious ovarian cancer (HGSOC) known to have deficient DNA damage and repair pathways. Datasets obtained from ovarian cancer (OC) cell lines treated with olaparib and chemotherapeutics as well as HGSOC patients’ cohort by analysing The Cancer Genome Atlas (TCGA) data, but also results from different clinical trials were analyzed. Upon determination of the homologous recombination repair deficiency (BRCAness) from exome sequencing data of HGSOC, a 24 gene signature separating BRCAness-positive from BRCAness-negative HGSOC samples was identified using machine learning and correlated to cytotoxic and T cell inflammatory signatures. The OS of patients was increased by BRCAness or CD8^+^ TILs. Poly-ADP ribose polymerase (PARP) inhibitors were shown to activate the type I IFN response and cGAS-STING pathway in OC cell lines. Furthermore, immune-related pathways were activated by BRCAness. Characterization of tumor immune cell infiltration in BRCA-ness demonstrated an association with suppressive tumor associated TREM2 macrophages expressing marker genes, such as LILRB4 and ITGB2, which were found to be downregulated by combination therapy.

Stefano Indraccolo (The Veneto Institute of Oncology, Padova, Italy) suggested liquid biopsies as a tool to monitor the therapeutic and adverse effects of immunotherapy in lung cancer with the advantage of minimal invasiveness and repeatability. Therefore, tumor heterogeneity and dynamic changes at the molecular level could be assessed. However, disadvantages of liquid biopsy include the short half-life and the very low concentration of circulating tumor (ct) DNA. Initially, the clinical feasibility and application of monitoring advanced NSCLC by genotyping of plasma during immunotherapy was shown by the identification of KRAS mutations in circulating free DNA (cfDNA) and their correlation with the risk of progression and death in this disease. Subsequently, NGS was used to track mutations in longitudinal liquid biopsies of immunotherapy treated patients. Comparison of liquid biopsies at baseline and early during treatment with ICPi could predict the probability for long-term clinical benefit. Integration of these results with PD-L1 expression and histology improved the prediction model and enabled to stratify the risk of patients for progression-free survival (PFS) and OS. These results are currently validated for their use for personalizing ICPi treatment. Importantly, the static and dynamic cfDNA quantification and genetic alterations were associated with the risk of detrimental effects, such as early death or hyper progressive disease following ICPi treatment, suggesting a cut-off definition and proposal for risk assessment in the clinical practice. Thus, analysis of liquid biopsies at early time points were highly informative and various molecular parameters were associated with detrimental effects including cfDNA concentrations, chromosomal instability and secretion of cytokines. In the future, omics-based analyses of liquid biopsies will likely be used as tools for precision medicine.

Stefan Glück (Miami, USA) gave an overview about the promise of IO in the future by providing information about the knowledge achieved over the last decade in cancer therapy from targeting tumor cells to targeting immune cells with the TME as a key factor in modulating immunotherapies. Since the responses to ICPi are limited in the various diseases, there is an urgent need for improvement of their efficacy and the selection of patients prior to therapy based on their disease immune profile. One possibility is the combination of ICPi with various standard therapies including chemotherapy and RT or with other ICPi, which leads to a better outcome and increased survival of patients as demonstrated in various clinical trials. However, novel immunotherapies are required to enhance the outcome of patients, such as novel ICPi directed against e.g. LAG-3, TIM3 and TIGIT, but also other strategies like CAR therapies with focus on CAR T or CAR NK cells. Since a major disadvantage of these therapies is to reach the lesions by their intra-venous application, direct tumor injections are currently an emerging technique for IO delivery. In order to improve IO therapies, the “cold” immune desert tumors should be converted into “hot” tumors. To revert primary and secondary resistances to tumor therapies and increased responses to cancer antigens, autologous approaches are required. Furthermore, IO interventions should move into precancer settings. Next to T cells and NK cells, macrophages and neutrophils as well as chemokines involved in the crosstalk between tumors and tumor-associated macrophages (TAM) became into focus as targets. An example was given by the chemokine receptor CCR-5, which is overexpressed in different cancer types. A monoclonal antibody (mAb) directed against CCR-5 was shown to block metastasis and enhance cell death obtained by DNA damaging chemotherapy. Thus, in the future, novel clinical (combinatorial) trials will become available and will include targeting of monocytes and TAM. In sum, both the efficacy as well as sustainability of T cell responses in immunotherapies should be enhanced, since anti-tumor effector T cells are often insufficiently activated.

Stina Wickström from the Department of Oncology/ Pathology of the Karolinska Institute in Stockholm, Sweden, reported on a clinical trial using a combination of adoptive T cell therapy (ACT) with a DC vaccine. The rational of this approach was based on the fact that tumor-infiltrating lymphocyte (TIL) therapy had a better progression-free survival (PFS) in anti-PD-1 refractory melanoma patients than therapy with ipilimumab. A two-step immunotherapy protocol compared ACT plus interleukin (IL)-2 to ACT plus IL-2 plus DC vaccine with T cell infusion followed lymphocyte depletion. The T cell receptor (TCR) repertoire was analysed prior and after therapy demonstrated a survival of T cell clones for more than 2 years, which could be also found at the vaccination side. In addition, the role of the reactive oxygen species (ROS), known to control and dampen the immune response thereby preventing tissue damage and inflammation, was determined regarding their effect on lymphocyte function. Tumors could use ROS thereby inactivating CD8^+^ CTLs and NK cells. Indeed, treatment with H_2_O_2_ for ROS induction resulted in a reduced NK cell-mediated lysis of K562 cells, while it increased intra-cellular ROS levels in NK cells. To protect NK cells, but also TIL from ROS damage in the TME, different compounds known to activate NRF2 were employed for the treatment of NK cells and TILs prior to H_2_O_2_ administration. Treatment with small molecules, in particular auranofin, resulted in resistance to ROS in NK cells and TILs thereby leading to an increased tumor elimination by NK cells, autologous TILs and CAR T cells. In sum, Stina Wickström gave insights into the protection of CD8^+^ cytotoxic T lymphocytes (CTLs) from damaging oxidative stress of the tumor microenvironment (TME) as well as a more efficient generation of tumor-specific T cells.

Next to the use of TILs for immunotherapy, Hinrich Abken (Center for Interventional Immunology, Regensburg, Germany) summarized the history of CAR T cell therapy by engineering T cells to produce proteins on their cell surface, called CARs. These CAR T cells recognize cancer cells in order to more efficiently target and destroy them. During the last decade, the generated CARs were modified in order to increase their efficacy. Based on the improvement of the cytolytic T cell activity, he presented data on CD30-CEA CAR T cells studied in a phase I clinical trial regarding dose-finding and feasibility in CRC patients with liver metastasis. Another approach in lymphoma demonstrated that CAR T cell killing was improved by blocking CD30/CD30L, but the position of the anti-CD30scFv within the CAR construct was important. Since for the maintenance of CAR T cell activity cytokine help is required, CAR T cells were engineered to secrete cytokines to activate innate cells for attacking cancer cells that are usually not recognized by CARs. An anti-CEA-CAR with an induced IL-12 was generated, but treatment failed in advanced disease. Therefore, a combination of CARs with induced IL-12 and inducible IL-18 was developed, which improved the survival of pancreatic cancer in mice due to the induction of NKG2D^+^ NK cells. These promising data resulted in the design of the 4th generation of CARs. A membrane anchored IL-18-TLR4-CD40 as adjuvant for CAR T cells, which amplified the cytokine release, was created. This was able to amplify the functional activity of CAR T cells and melanoma TILs leading to an enhanced cytotoxicity against HLA-A2-matched melanoma cells i*n vitro*. Furthermore, the IL-12 was integrated into the CAR exodomain and these IL-12-CAR T cells display a NK cell signature. Since the CAR T cell activity could be repressed by TGF-*β*1, an artificial switch receptor was developed leading to resistance to TGF-*β*1 and thus overcome the TGF-*β* repression. Based on these results, CARs become chic and cytokine help has demonstrated to intensify CAR T cell activity and sustainability.

Michael Bachmann (Helmholtz Center Dresden, Germany) talked about the nuclear auto-antigen (La) known to be involved in different autoimmune diseases, which against anti-La mAbs were generated, which are currently used in modular bi-specific antibody (UniMAB) as well as adaptor CAR platforms (UniCARs, RevCARs). The modular bi-specific antibody platform (UniMAB) was designed to accelerate the development of conventional bi-specific Abs including the two CD3-CD33 and CD3-PCSA bi-specific Abs, which were employed in the first clinical trial in 2018. In addition, a first modular CAR system, called UniCAR with comparable activity to the conventional CARs, was generated for CAR T and CAR NK cell approaches to reduce their risks, such as the cytokine release syndrome as well as off target effects. Besides the possibility to regulate the activity of UniCAR T cells via application of target molecules, this modular concept is highly flexible and allows the targeting of multiple tumor species. For this purpose, a series of target modules were developed for targeting various leukemic cells and solid tumors. First clinical trials targeting AML and prostate cancer are currently running showing proof of concept for both the functionality and switchability of the UniCAR platform. In addition, theranostic target molecules were developed for imaging of immunotherapy and combining immunotherapy with radionuclide therapy. Based on the UniCAR platform a further switchable adaptor CAR platform termed RevCAR was established. The reduced size of RevCAR genes allowed the construction of viral vectors containing more than one CAR gene, which is the prerequisite for a reliable use of these synthetic receptors. Like the UniCAR system, the modular adaptor RevCAR system is switchable, universal, flexible and programmable and has a high efficient killing activity of tumor cells in an antigen-specific and Rev-dependent manner. It is controllable by a switch on and off system thereby improving the safety. Flexible targeting of multiple antigens using an universal RevCAR T cell was possible thereby overcoming immune escape and tumor antigen heterogeneity. It further allowed a combinatorial targeting to increase tumor specificity. Next to targeting tumor cells by antigens and ICPi, the TME could be also targeted as demonstrated by using the fibroblast activation protein (FAP) as proof of principle. FAP-expressing cells could be infiltrated and specifically killed by UniCAR T cells suggesting the accumulation of an anti-FAP TME with an immunotherapeutic effect in vivo. In sum, M. Bachmann developed various immunotheranostic platforms, which are currently used for the treatment of patients, but could also be used for diagnostics, imaging as well as for targeting the immune suppressive TME and thus might improve the personalized therapy. The increased safety profile might also allow the application of these platforms for other diseases, e.g. infectious diseases.

Martin Bornhäuser from the University Hospital of the TU Dresden, Dresden, Germany summarized the current limitations of the different immunotherapeutic approaches developed for the treatment of AML. These included unspecific approaches, like IFN, IL-2 and allogenic cell therapy, but also DC-based/ mRNA-based vaccination of drug-conjugated Abs, ICP modulation, but also the use of BITEs as well as genetically engineered CAR NK and T cells. However, AML have often lost or downregulated HLA class I antigens, so the challenge is to tackle the immune invasion of AML. So far, a number of targets are under investigation for the treatment of AML patients, such as iCP molecules as well as specific antigens expressed on AML. Different approaches were successfully employed, like the use of toll-like receptor (TLR)7/8 matured DC vaccines, a novel ICPi targeting CD47 as well as the combination treatment of anti-CD47 Ab with demethylating agents. Furthermore, a two component CAR T cell platform, named UniCAR, was used for targeting the CD123 antigen, which was safe and efficient. Mesenchymal stroma cells (MSCs) as well as anti-leukemic effector cells were targeted within the bone marrow TME using activating UniCAR T cell inflammatory stimuli with the expression of ICP and lymphocytic adhesion molecules in MSCs. This approach significantly altered the transcriptome of MSCs as demonstrated by RNAseq analysis. Transcriptome analysis revealed that MSCs induced senescence of unmodified T cells as characterized by the loss of CD28 and gain of CD57. In addition, these MSCs interfered with the proliferative and inflammatory capacity of CD123-targeting UniCAR T cells by affecting their cytotoxicity. L patients by targeting MSC or leukemic effector cells.

Angus Dalgleish from the St George's University of London, London, Great Britain, gave an overview about the role of innate immune system in cancer control. He demonstrated a survival benefit in Mycobacterium vaccine-treated patients with lung adenocarcinoma, which have an increased survival compared to chemotherapy alone. More recently, an improved agent based on Mycobacterium obuense known as (IMM-101) statistically improved PFS and OS in metastatic cancer patients when given with gemcitabine vs gemcitabine alone. He reported that *γδ* T cells were activated by killed Mycobacteria, which was mediated by cytokines produced by mature DCs leading to an increased cytotoxicity to susceptible target cells. Furthermore, activated *γδ* T cells were able to recruit adaptive immune responses. High expression of granulysin was found in NK cells, medium expression in *γδ* T cells and low expression in *αβ* T cells and was released by *γδ* T cells upon stimulation with mycobacteria. Furthermore, granulysin induced DC activation. Interestingly, the different expanded *γδ* T cells had distinct ICP profiles compared to the *αβ* TCR population as well as NK cells. Thus, ICPi were more effective in the presence of the mycobacteria vaccine activating NK cells and mature DCs. Using the vaccine IMM-101, patients showed a clinical response in combination with ipilimumab. In combination, the synergy might be due to the activation of NK cells in MDC as well as the unique activation of *γδ* T cells. Thus, targeting unique ICP and *γδ* T cells, such as NKG2D, as well as combinations of IMM-101, NKG2D and pembrolizumab have to be explored in future studies.

The talk of Bernard A. Fox from the Earle A. Chiles Research Institute in Portland, Oregon, USA, discussed the discovery of novel non-canonical alternative neo-antigens in cancer and their use in a clinical trial. In the last three years, novel peptides were identified on the surface of cancer cells, which were derived from 98% of the non-expressed genome, and many appeared to be only expressed by cancer cells. Since these peptides were not expressed in the thymus, they represent non-mutated cancer neo-antigens, some of which appear to be epigenetically regulated and associated with a worse patients’ outcome. These peptidomes not available in the uni-prot database of expressed genes were identified by employing whole transcriptomic databases to evaluate spectra obtained by mass spectroscopic analysis of peptides eluted from HLA of cancer cells. The discovery of novel non-canonical proteins has expand the cancer immunopeptidome and represents a new class of neo-antigens that in some cases appears to be shared by many cancer types. Since they are short-lived and apparently unable to prime immune responses in vivo, these neoantigens were not b identified earlier. Vaccines enriched for short-lived proteins (SLiPs) containing tumor-associated antigens and non-canonical proteins could induce a cross-protective immune responses against SLiPs via a P62-dependent mechanism. Furthermore, a combination of this SLiPs vaccine with anti-GITR and anti-PD-1 mAb increased the therapeutic efficacy in preclinical models leading to a clinical trial. This trial combined DPV-001, an off-the-shelf vaccine, with anti-PD-1 ± anti-GITR for patients with head and neck squamous cell cancer (HNSCC). In depth immunomonitoring was performed using both biopsies as well as peripheral blood. Flow cytometric analysis demonstrated that the vaccine alone could induce activated CD4^+^ and CD8^+^ effector memory T cells. Preliminary multiplex immunofluorescence and scRNAseq analysis of biopsies demonstrated an increase in TIL in all patients with two patterns of clonal TCR expansion in the tumor, with the proliferation of previously undetected clones as well as the expansion of existing clones. These TIL exhibited increased expression of IFN-γ and granzyme, but also of ICP, such as LAG3 and TIM-3.

Markus Maeurer from the Champalimaud Centre for the Unknown in Lissabon, Portugal, reported about the relevance of tumor heterogeneity for the design and efficacy of immunotherapies. He pointed out that the characterization of biological and clinical relevant parameters by tissue array analyses of the local and systemic immune responses could decipher the intratumoral variation defined by (i) focal differences in T cells with different TCR repertoires, (ii) epigenetic imprints leading to differential cytokine production of TIL directed against nominal tumor-associated antigens, (iii) differences in the mutational landscape and (iv) a diverse immune cell infiltrate defined by TCR alpha/beta, TCRgamma/delta and MAIT cells. M. Maeurer developed a pipeline for the in depth analysis of TILs by applying different omics-based technologies using PDAC, CRC and cholangiocarcinoma as a model. These results might allow to develop therapies with increased specificity, enhanced target reactivity and long-term anti-tumor reactivity using a broader array of immune effector cells restricted by classical (MHC class I/II) or non-classical (CD1 and MR1) MHC molecules. A number of risk factors and therapy resistance mechanisms were identified in tissue specimen from primary PDAC or CRC by analysing TILs in regard to the quality of the cytokine production, the breadth of the TCR repertoire and their spatial distribution within the TME. A heterogeneous expression pattern of IFN-γ and IL-17 was found in tumors, which correlated with the expression of KRAS, MUC4, mesothelin or tumor-associated antigens with CD3^+^ T cells producing IFN-γ mainly found in tertiary lymphoid structures (TLS). Based on this information, TILs were expanded using an established protocol in order to avoid IL-17 producing areas. The expanded TILs were able to recognize private mutations of patients, which were defined by IFN-γ production. Next to *αβ* TCR-positive cells, CD1-restricted *γδ* T cells were expanded. In summary, M. Maeurer provided data regarding a robust clinical grade expansion of T cells from gastro-intestinal malignancies, which strongly depended on the location of T cells, their quality and the nature of response. These TILs secreted Th1 cytokines, CXCL9 and CXL10, which allowed an increased invasion of TILs into tumor tissue. Furthermore, these TILs had a memory phenotype associated with clinical responses, a composite of *αβ* and *γδ* T cells as well as tumor-reactive MAIT cells. There existed a molecular blueprint of successful immune responses, which could be manipulated in order to enhance immune effector functions. The efficacy of cellular immunotherapies might be increased by molecular strategies favoring Th1 responses, while downregulating Th2/TH17 responses in association with increased survival of T cells in hypoxic tissue. The design of CARs with the ability to upregulate chemokine receptors might also facilitate T cell trafficking and increase T cell fitness and survival of CAR T cells in a composite T cell therapy approach with the aim to increase MHC expression on tumor cells and to downregulate immune suppressive factors elaborated by non-transformed cells–or by tumor-associated pathogens–in the TME.

Overall, the conference attendees experienced different presentations ranging from short talks, posters, symposium talks and a keynote lecture at the cutting edge of tumor immunology and immunotherapy with their potential and challenges. Due to the optimal size of TIMO, attendees had the opportunity for extensive collaboration between each other. This will drive the field in the future.

## Data Availability

Not applicable, since this is a meeting report.

## Abbreviations

Ab: Antibody
ACT: Adoptive cell therapy
AHR: Arylhydrocarbon receptor
AI: Artificial intelligence
ALL: Acute lymphatic leukemia
AML: Acute myeloid leukemia
APC: Antigen presenting cells
APM: Antigen processing machinery
Arg1: Arginase-1
*β*_2_-*m*: Beta-2-microglobulin
BA: Bile acid
BET: Bromodomain and extraterminal motif protein inhibitor
BC: Breast cancer
BGN: Biglycan
BITE: Bi-specific T cell engager
CAR: Chimeric antigen receptor
cfDNA: Circulating free DNA
ctDNA: Circulating tumor DNA
CTLA-4: Cytotoxic T lymphocyte-associated protein 4
CTL: Cytotoxic T lymphocyte
CyToF: Cytometry by Time of Flight
DAC: 5-Aza 2 ‘-deoxycytidine
2DC: Dendritic cells
DLBCL: Diffuse large B cell lymphoma
EGF-R: Epidermal growth factor receptor
F.: Fusobacterium
FAP: Fibroblast activation protein
FDA: Federal drug agency
FXR: Farneosoid receptor
GI: Gastrointestinal
GM-CSF: Granulocyte–macrophage colony-stimulating factor
GMP: Good medical practice
GVHD: Graft versus host disease
HC: Heavy chain
HCT: Hematopoietic cell transplantation
HGSOC: High grade serious ovarian cancer
HIF: Hypoxia inducible factor
3-HKA: 3-Hydroxy-L-kynurenamine
HLA: Human leukocyte antigen
HNSCC: Head and neck squamous cell cancer
HR: Hormone receptor
HSCT: Hematopoietic stem cell transplantation
ICB: Immune checkpoint blockade
ICD: Immunogenic cell death
ICP: Immune checkpoint
ICPi: Immune checkpoint inhibitor
IDO: Indolamine 2,3-deoxygenase
IED: Immune effector dysfunction
IFN: Interferon
IHC: Immunohistochemistry
IL: Interleukin
ILC: Innate lymphoid cell
IMC: Imaging mass cytometry
iNOS: Inducible nitric oxide species
IO: Immune oncology
KIR: Killer Ig-like receptor
kyn: Kynurenine
mAb: Monoclonal antibody
MAIT: Mucosal-associated invariant T cell
MDSC: Myeloid-derived suppressor cell
miRNA: MicroRNA
MMR-d: Mismatch repair deficient
MMR: Mismatch repair
MMR-p: Mismatch repair proficient
MSC: Mesenchymal stromal cells
NAD: Nicotinamide adenine dinucleotide
NET: Neutrophil extracellular trap
NGS: Next generation sequencing
NKG2D: Natural killer group 2D
NKG2D-L: Ligand of NKG2D
NP: Nanoparticle
NSCLC: Non-small cell lung carcinoma
OC: Ovarian cancer
OR: ODD ratio
OS: Overall survival
PAP: Prostatic acid phosphatase
PBL: Peripheral blood lymphocytes
PDAC: Pancreatic ductal adenocarcinoma
PD1: Programmed death receptor 1
PD-L1: Programmed death ligand 1
PEI: Polyethylenimine
PFS: Progression-free survival
PDX: Patient-derived tumor xenografts
RCC: Renal cell carcinoma
RNAseq: RNA sequencing
RR: Response rate
ROS: Reactive oxygen species
RT: Radiation therapy
scRNA seq: Single cell RNA sequencing
siRNA: Small interfering RNA
SLIP: Short lived protein
SLN: Sentinel lymph node
TAM: Tumor associated macrophage
TAN: Tumor associated neutrophil
TCGA: The Cancer Genome Atlas
TCR: T cell receptor
TEM: T effector memory cell
TF: Transcription factor
TIL: Tumor infiltrating lymphocyte
TKI: Tyrosine kinase
TLR: Toll-like receptor
TLS: Tertiary lymphoid structure
TME: Tumor microenvironment
TNBC: Triple negative breast cancer, Treg, regulatory T cell
TRUCK: T cell-redirected for antigen-unrestricted cytokine-initiated killing

